# A longitudinal study of parent–child relationship and behavioral problems in children aged 3 to 6: the mediating role of screen time

**DOI:** 10.3389/fpsyg.2026.1794353

**Published:** 2026-05-25

**Authors:** Xiaojun Ling, Mengqi Li, Jiahui Sun

**Affiliations:** 1 School of Education Science, Nantong University, Nantong, China; 2 School of Teacher Education, Daqing Normal University, Daqing, China

**Keywords:** behavioral problems, children, longitudinal study, parent–child relationship, screen time

## Abstract

In the context of the digital era, screen media has become deeply embedded in family life, reshaping traditional parent-child interactions and altering the developmental ecology of early childhood. To examine how these dynamics unfold over time, we conducted a longitudinal study involving 532 Chinese children aged 3 to 6 years, assessed across three waves spaced 4 months apart. Analyses revealed that conflictual parent-child relationships at Time 1 significantly predicted both externalizing and internalizing behavioral problems at Time 3, with screen time at Time 2 serving as a partial mediator. In contrast, close parent-child relationships at Time 1 did not significantly predict later screen time but were directly associated with lower levels of externalizing behavioral problems at Time 3.

## 1 Introduction

In many countries, screen media has become deeply embedded in children's daily lives and learning experiences (Kaur et al., 2019). While screen media provides children with diverse opportunities for learning and entertainment, excessive exposure has increasingly attracted scholarly and public concern due to its potentially detrimental impact on child development (Lindsay et al., 2019; Hawi et al., 2019). Screen media exposure among young children has shown both earlier onset and excessive duration. A substantial proportion of children aged 3 to 6 worldwide exceed the World Health Organization's recommendation of limiting daily screen exposure to 1 h (Durham et al., 2021; Kim et al., 2022; Cliff et al., 2017; Chaput et al., 2017; Vale and Mota, 2020; Gao et al., 2022; Frata et al., 2021). In particular, a study conducted in China revealed that excessive screen use is prevalent among children aged 4–6 years (Teng et al., 2019). Extensive empirical research has identified prolonged screen time as a significant risk factor for the emergence of behavioral problems in children (Likhitweerawong et al., 2024; Yang et al., 2025). Additionally, a substantial body of research has established the parent-child relationship as a robust predictor of children's behavioral problems (Torjinski and Horwood, 2023).

In the context of the new era of digital parenting, the parent-child relationship not only directly shapes children's behavioral development but may also exert an indirect influence by affecting the duration, content, and patterns of their screen media use (Wong et al., 2021; Lu and Li, 2022). Although prior research has primarily examined the dyadic relationships between parent-child dynamics and behavioral problems or between screen media exposure and behavioral problems, systematic investigations that integrate the three constructs into a unified explanatory framework remain notably scarce. Focusing on preschool children aged 3 to 6, this study adopts a longitudinal design to explore the distinct mediating pathways through which close vs. conflictual parent-child relationships influence children's behavioral problems, specifically through patterns of screen media use. The findings are expected to offer robust empirical support for guiding the development of early family-based screen media intervention strategies.

## 2 Literature review

### 2.1 The association between the parent-child relationship and children's behavioral problems

The parent-child relationship is a relatively stable relational dynamic shaped through sustained emotional, physical, and behavioral interactions. A high-quality parent-child relationship provides essential support for children's developmental well being and plays a foundational role in shaping early socialization and adaptive functioning (Briegel et al., 2019). The parent-child relationship is commonly conceptualized along the dimensions of relational closeness and conflict. Closeness reflects warmth, acceptance, and mutually responsive interactions, whereas conflict is marked by frequent criticism, disagreement, rejection, neglect, and, in some cases, physical confrontation (Savell et al., 2023).

The quality of the parent–child relationship is widely regarded as a key predictor of children's behavioral problems (Schuiringa et al., 2015). A close and supportive parent–child relationship is associated with more adaptive behavioral and emotional functioning in children, whereas parent–child conflict is consistently linked to elevated levels of both externalizing problems (e.g., aggression, inattention) and internalizing problems (e.g., anxiety, depression) (Lau et al., 2006; Coe et al., 2024). Frequent conflict and emotional invalidation are more saliently predictive of externalizing behavioral problems, whereas diminished emotional closeness is more consistently associated with internalizing difficulties, including anxiety, depression, and social withdrawal (Acar et al., 2019; Kretschmer et al., 2016).

Cross-cultural research has provided additional evidence for this pattern. In Western contexts, early behavioral problems in children are strongly predicted by harsh parental criticism and elevated parent–child conflict, while emotional detachment and high parental control are consistently associated with both social withdrawal and aggressive tendencies (Hillekens et al., 2020). In Chinese family contexts, both parental detachment and reduced relational closeness have been shown to significantly predict children's behavioral problems (Wang et al., 2025; Sun et al., 2024). Collectively, these findings underscore the critical role of parent–child relational dynamics in the emergence of children's behavioral problems across diverse cultural contexts.

Although a substantial body of empirical research has explored the association between the parent–child relationship and children's behavioral problems, notable limitations persist—most notably, the predominant use of cross-sectional designs, which constrain the ability to examine dynamic relationships and temporal associations that may underlie observed patterns. In response to these limitations, the present study employs a longitudinal design to investigate their temporal interplay.

### 2.2 The mediating role of screen time between parent-child relationship and problem behaviors

Children's screen exposure is shaped by multiple ecological factors, with the home environment—particularly the quality of the parent-child relationship—emerging as a primary determinant of screen time, media content, and usage context among children aged 3 to 6 (Niu et al., 2020). Empirical evidence indicates that a high-quality parent–child relationship effectively reduces the risk of excessive screen exposure (Schmidt-Persson et al., 2024; Çaylan et al., 2021). Positive emotional support and frequent parent–child communication during media use foster children's media literacy, strengthen cognitive control, and enhance emotional regulation, thereby decreasing their susceptibility to screen dependence (Meri et al., 2023; Wu et al., 2023). Conversely, negative parent–child relationships increase the likelihood of excessive screen exposure. Maternal rejection and neglect, for instance, are closely linked to prolonged screen time in children (Erat Nergiz et al., 2020; Zhang et al., 2024). It is worth noting that most studies examining the parent-child relationship and children's screen time have been conducted in Western contexts, where consultative parent-child interactions and parental mediation strategies are emphasized (Schwarzer et al., 2022). By contrast, Eastern cultural contexts often highlight attachment, obedience, and parental authority. Within this framework, parents frequently act as media gatekeepers, relying more heavily on restrictive mediation strategies (Geng et al., 2023). These cultural distinctions underscore the need for further investigation into how parent–child dynamics shape screen time in non-Western contexts, particularly within Chinese families.

Excessive screen time has been associated with a range of physical health risks, such as visual impairment, reduced sleep quality, and increased obesity rates, as well as with heightened risks of both externalizing and internalizing behavioral problems (Neshteruk et al., 2021; Liu et al., 2021). Behavioral problems are defined as maladaptive patterns of behavior that deviate from developmental norms and impair social functioning, typically categorized into externalizing (e.g., aggression, impulsivity) and internalizing (e.g., anxiety, withdrawal) domains (Takahashi et al., 2024). Evidence suggests that excessive screen time is associated with higher levels of aggression, oppositional conduct, hyperactivity (Tamana et al., 2019; Gialamas et al., 2020), as well as symptoms of anxiety, depression, and social withdrawal (Saleem et al., 2024; Zhu et al., 2023). The influence of screen time on behavioral problems is characterized by three features. First, a dose-response effect has been observed: longer screen time predicts greater risk (Gülbetekin and Yildirim, 2023; AlSamhori et al., 2025). Second, the effect is broad-based, influencing both externalizing and internalizing behaviors (Eirich et al., 2022; Neville et al., 2021). Third, early and prolonged screen exposure appears particularly detrimental, showing stronger predictive effects on later behavioral problems (McArthur et al., 2020). Nevertheless, some studies caution that the temporal ordering of this association remains unclear, as screen time may also reflect underlying family or individual vulnerabilities (Ophir et al., 2021; Jörren et al., 2023). While substantial research has explored screen time and behavioral outcomes in adolescents, evidence concerning younger children remains limited. Early childhood (ages 3–6) is a critical developmental window for shaping emotional regulation and behavioral patterns. At this stage, children typically lack sufficient self-regulation and discernment for healthy media use (Chia et al., 2022). Given the potential mediating role of screen time between parent–child relationship and problem behaviors, further investigation is urgently needed to clarify these pathways, particularly in cultural contexts such as China.

### 2.3 Research questions

In summary, while extensive research has examined the interrelations among the parent–child relationship, screen time, and children's behavioral problems, few empirical studies have incorporated screen time into integrative models that link parent–child dynamics to behavioral outcomes, particularly in early childhood and within longitudinal frameworks (Sampasa-Kanyinga et al., 2020). The rapid advancement of the digital era has led to the deep integration of smart screen media into family life, transforming the structure and dynamics of parent–child interactions and reshaping the proximal developmental context for children's physical and psychological growth. In this shifting landscape, children's screen time may serve as a key contextual factor that alters the pathways linking parent-child relationship quality to behavioral outcomes. Building on this framework, the present study proposes the following hypotheses:

1. Variations in parent–child relationship types significantly predict behavioral problems among children aged 3 to 6.
2. Different types of parent–child relationships are closely associated with children's screen time.
3. Children's screen time is positively associated with behavioral problems.
4. Screen time mediates the associations between parent–child relationship types and children's behavioral problems.

## 3 Materials and methods

### 3.1 Participant and procedures

This study employed a longitudinal design involving parents of children aged 3 to 6 years in Fuyang City, Anhui Province, China. A simple random sampling procedure was used to recruit 619 children and their mothers at Time 1 (T1), who completed the baseline assessment. Data were collected at three time points: T1 (*n* = 619), T2 (*n* = 588), and T3 (*n* = 532). Attrition occurred across waves, with 31 participants lost between T1 and T2 and 56 participants lost between T2 and T3, resulting in a total attrition of 87 participants (14.1%) across the study period. Attrition analyses comparing participants who remained in the study and those who dropped out on key baseline variables revealed no significant differences. Missing data due to attrition were handled using full information maximum likelihood (FIML) under the missing-at-random (MAR) assumption within structural equation modeling. To ensure data quality, responses with completion times below 2 min (based on pilot testing and observed response distributions) were excluded (*n* = 11). The final analytic sample consisted of 532 valid cases. All questionnaires were completed by mothers, who served as the primary caregivers.

The final sample comprised 289 boys (54.3%) and 243 girls (45.7%). The mean age of the children was 3.99 years (SD = 0.85). Specifically, 198 children were 3 years old (37.2%), 150 were 4 years old (28.2%), and 184 were 5 years old (34.6%). Table 1 provides a detailed overview of the participants' demographic information. All participants provided written informed consent prior to completing the questionnaire. The study was reviewed and approved by the Academic Ethics Committee of the School of Educational Science at Nantong University (Approval No. NT20231109).

**Table 1:** Descriptive characteristics of the sample.

Characteristics	n (%)/M (SD)
Gender	
Boys	289 (54.3%)
Girls	243 (45.7%)
Age (years)	3.99 (0.85)
Age 3	198 (37.2%)
Age 4	150 (28.2%)
Age 5	184 (34.6%)
Father's educational attainment	
High school or below	107 (20.1%)
Associate degree	202 (38.0%)
Bachelor's degree	200 (37.6%)
Master's degree	21 (3.9%)
Doctoral degree	2 (0.4%)
Mother's educational attainment	
High school or below	87 (16.4%)
Associate degree	164 (30.8%)
Bachelor's degree	256 (48.1%)
Master's degree	24 (4.5%)
Doctoral degree	1 (0.2%)
Father's occupation	
Unskilled/semi-skilled workers	104 (19.5%)
Skilled workers	149 (28.0%)
General civil servants	168 (31.6%)
Professional or mid-level administrators	107 (20.1%)
Senior professionals or administrators	4 (0.8%)
Mother's occupation	
Unskilled/semi-skilled workers	167 (31.4%)
Skilled workers	91 (17.1%)
General civil servants	172 (32.3%)
Professional or mid-level administrators	99 (18.6%)
Senior professionals or administrators	3 (0.6%)

### 3.2 Materials

#### 3.2.1 Assessment of parent–child relationship

The quality of the parent–child relationship was assessed using the Child-Parent Relationship Scale (CPRS; Pianta, 1992), a widely validated instrument grounded in attachment theory and relational models of early childhood development. The Chinese revised version of the CPRS, translated and adapted by Deng (2013), was employed in the present study. This version demonstrated satisfactory construct validity, as evidenced by confirmatory factor analysis (RMSEA = 0.058, CFI = 0.932, GFI = 0.907). The CPRS comprises two subscales: Closeness (7 items; e.g., “There is a warm, affectionate relationship between my child and me.”) and Conflict (8 items; e.g., “My child and I always seem to be struggling with each other.”). All items were rated by mothers using a 5-point Likert scale ranging from 1 (not at all true) to 5 (completely true), with higher scores indicating higher levels of perceived emotional closeness or relational conflict. Subscale scores were treated as continuous variables in the analyses. In the present sample, the scale demonstrated acceptable to good internal consistency, with Cronbach's α = 0.882 for the Closeness and Cronbach's α = 0.895 for the Conflict.

#### 3.2.2 Assessment of screen time

Children's screen time was measured through caregiver reports based on two items: (1) “On average, how much time per day does your child spend using electronic devices on weekdays (Monday to Friday)?” and (2) “On average, how much time per day does your child spend using electronic devices on weekends (Saturday and Sunday)?” The use of parent-reported screen time is a widely accepted approach in the existing literature for assessing children's exposure to electronic media (Zimmerman et al., 2007; Yalçin et al., 2021; Zhu et al., 2024). To calculate overall daily screen time, a weighted average was computed using the following formula: Average screen time = (weekday average × 5 + weekend average × 2)/7 (Chen et al., 2020). This method accounts for differences in daily routines between weekdays and weekends.

#### 3.2.3 Assessment of behavioral problems

Externalizing behavioral problems (EBP) and internalizing behavioral problems (IBP) were measured using the Strengths and Difficulties Questionnaire (SDQ; Goodman, 2001). The SDQ is a widely used, brief behavioral screening tool that has demonstrated good construct validity for identifying behavioral problems in preschool-aged children (RMSEA = 0.078, CFI = 0.912, TLI = 0.931). The SDQ uses a 3-point Likert scale, with responses ranging from 1 (not true) to 3 (certainly true). Externalizing behaviors problems were assessed using two subscales: Hyperactivity/Inattention (e.g., “The child is restless, overactive, and cannot stay still for long”) and Conduct Problems (e.g., “The child often fights with other children or bullies them”). Internalizing behaviors problems were assessed using the Emotional Symptoms subscale (e.g., “The child has many worries and often seems worried”) and the Peer Problems subscale (e.g., “The child has at least one good friend”). Each subscale consists of 5 items. The SDQ demonstrated acceptable internal consistency in the present study, with Cronbach's α = 0.891 for externalizing behaviors problems and Cronbach's α = 0.853 for internalizing behaviors problems.

#### 3.2.4 Covariates

Several covariates were controlled for in the study, including the child's gender and family socioeconomic status (SES). SES was operationalized by combining parental education and occupation levels. Specifically, parental education was categorized into six levels: (1) primary school or below, (2) junior high school, (3) senior high school, vocational school, or technical school, (4) junior college, (5) bachelor's degree, and (6) postgraduate degree or above. Parental occupation was classified into five levels according to the Hopkins Occupational Classification: (1) non-technical and semi-skilled workers, (2) skilled workers, (3) general administrative personnel, (4) professionals and mid-level administrative personnel, and (5) senior professionals and senior administrators. Following the SES synthesis approach proposed by Ren (2010), principal component analysis (PCA) was conducted using four standardized indicators, including father's education, mother's education, father's occupation, and mother's occupation. The first principal component was retained to construct the SES index, accounting for 61.09% of the total variance.

Family SES was computed as a weighted composite score based on the factor loadings of the first principal component: Family SES = (0.792 × father's education + 0.836 × mother's education + 0.728 × father's occupation + 0.767 × mother's occupation)/0.6109. Higher scores indicate higher levels of family socioeconomic status.

### 3.3 Data analysis

Data were collected online via the Wenjuanxing platform, with all questionnaire items set as mandatory to ensure data completeness and the absence of missing values. Preliminary analyses, including descriptive statistics, Pearson correlations, and one-way analysis of variance (ANOVA), were conducted using SPSS 26.0 to examine basic patterns among variables. To test the hypothesized mediation model, longitudinal structural equation modeling was performed in Mplus 8.0 using maximum likelihood estimation combined with the bootstrap method, which was employed to derive bias-corrected confidence intervals. While this approach does not strongly rely on the assumption of multivariate normality, normality tests were nonetheless conducted for key variables as part of a broader data quality assurance procedure (Ghasemi and Zahediasl, 2012). Skewness and kurtosis statistics for all study variables indicated acceptable normality. Specifically, Closeness (−1.12, 1.19), Conflict (0.82, 0.43), Screen Time (1.84, 2.17), Peer Problems (0.50, −0.12), Emotional Symptoms (1.85, 3.16), Conduct Problems (0.30, −0.19), and Hyperactivity/Inattention (0.22, −0.23) fell within a range suggesting approximate normal distribution.

## 4 Results

### 4.1 Common method bias control

Given the exclusive use of maternal self-report data, the potential for common method bias (CMB) was addressed through multiple strategies. First, a three-wave longitudinal design with 4-month intervals between assessments was employed to reduce source and timing-related biases (Conway and Lance, 2010). Second, structural equation modeling explicitly accounted for the relationships between latent constructs and measurement error, minimizing bias at the model level (MacKenzie and Podsakoff, 2012). Finally, Harman's single-factor test, conducted at Time 1, revealed that the first unrotated factor explained 27.95% of the variance—well below the conventional 40% threshold indicative of problematic CMB.

### 4.2 Descriptive statistics and correlations

Descriptive statistics indicated that scores for screen time, conflictual parent–child relationship, and internalizing behavioral problems were slightly below the scale midpoint, whereas close parent-child relationship and externalizing behavioral problems were above the midpoint (see Table 2).

**Table 2:** Descriptive statistics and bivariate correlations between the key variables.

Vairables	M	SD	1	2	3	4	5	6	7
CPCR	4.553	0.446	–						
ConPCR	1.93	0.694	−0.399^***^	–					
ST	1.17	1.039	−0.077^***^	0.206^***^	–				
PP	2.143	0.357	−0.041^***^	0.245^***^	0.077^*^	–			
ES	1.243	0.419	−0.015^***^	0.260^***^	0.161^***^	0.262^***^	–		
CP	2.624	0.333	−0.048^***^	0.195^***^	0.106^**^	0.086^**^	0.205^***^	–	
HI	1.799	0.269	−0.255^***^	0.245^***^	0.143^***^	0.091^**^	0.237^***^	0.252^***^	–

CPCR, close parent-child relationship; ConPCR, conflictual parent-child relationship; ST, screen time; PP, peer problems; ES, emotional symptoms; CP, conduct problems; HI, hyperactivity/inattention.

^***^p < 0.001; ^**^p < 0.01; ^*^p < 0.05.

Pearson correlation analyses revealed several significant associations. Screen time was negatively associated with close parent-child relationship (*r* = −0.077, *p* < 0.001), and positively associated with conflictual parent–child relationship (*r* = 0.206, *p* < 0.001), peer problems (*r* = 0.077, *p* < 0.05), emotional symptoms (*r* = 0.161, *p* < 0.001), conduct problems (*r* = 0.106, *p* < 0.01), and hyperactivity/inattention (*r* = 0.143, *p* < 0.001).

Moreover, close parent–child relationship was negatively correlated with conflictual parent-child relationship (*r* = −0.399, *p* < 0.001), peer problems (*r* = −0.041, *p* < 0.05), emotional symptoms (*r* = −0.015, *p* < 0.001), conduct problems (*r* = −0.048, *p* < 0.01), and hyperactivity/inattention (*r* = −0.255, *p* < 0.001). Conflictual parent–child relationship was positively associated with peer problems (*r* = 0.245, *p* < 0.001), emotional symptoms (*r* = 0.260, *p* < 0.001), conduct problems (*r* = 0.195, *p* < 0.001), and hyperactivity/inattention (*r* = 0.245, *p* < 0.001). Lastly, peer problems were significantly correlated with emotional symptoms (*r* = 0.262, *p* < 0.001), conduct problems (*r* = 0.086, *p* < 0.01), and hyperactivity/inattention (*r* = 0.091, *p* < 0.01). Emotional symptoms were also significantly correlated with conduct problems (*r* = 0.205, *p* < 0.001) and hyperactivity/inattention (*r* = 0.237, *p* < 0.001). Conduct problems were also significantly correlated with hyperactivity/inattention (*r* = 0.252, *p* < 0.001).

### 4.3 Group differences in behavioral problems

Group differences in behavioral problems among children aged 3 to 6 were examined using independent t-tests and one-way analyses of variance (ANOVAs) to assess the effects of gender, age, and family socioeconomic status (SES). No significant differences were found based on gender or age. However, a one-way ANOVA revealed a significant effect of family SES on both internalizing behavioral problems (*F* = 3.741, *p* < 0.05) and externalizing behavioral problems (*F* = 3.198, *p* < 0.05). Post hoc comparisons using the Tukey HSD test indicated that children from low-SES families exhibited significantly more internalizing behavioral problems than those from high-SES families (*p* < 0.05). Similarly, children from low-SES families showed significantly more externalizing behavioral problems than their high-SES counterparts (*p* < 0.05). No significant differences were observed between children from middle-SES and high-SES families (*p* > 0.05), or between those from middle-SES and low-SES families (*p* > 0.05).

### 4.4 Associations between parent–child relationship and behavioral problems

**Figure 1:**
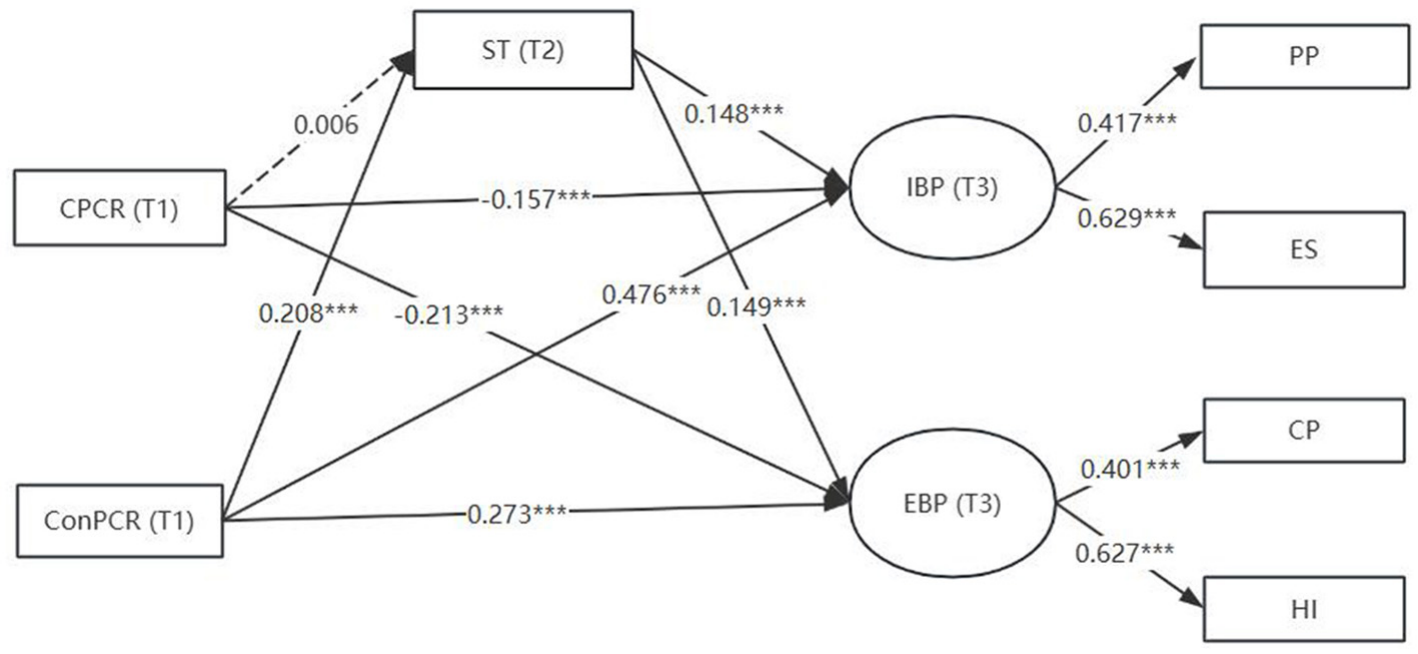
Structural relationships among parent–child relationship, screen time, and behavioral problems.

Structural equation modeling was conducted to examine the associations between parent-child relationship quality and preschool children's behavioral problems. Specifically, close and conflictual parent-child relationships were included as predictors, screen time was specified as a mediator, and internalizing and externalizing behavioral problems were modeled as outcome variables. The results of model comparison indicated that the partial mediation model provided a significantly better fit to the data than the full mediation model (Δχ^2^(2) = 15.30, *p* < 0.001). In addition, modification indices were inspected; however, no additional paths that lacked theoretical justification were added, in order to avoid overfitting and to preserve model parsimony. The final model demonstrated an acceptable fit to the data: χ^2^(7) = 19.27, *p* < 0.05; RMSEA = 0.057, 90% CI [0.028, 0.089]; CFI = 0.950; TLI = 0.917; and SRMR = 0.028. Standardized path coefficients are presented in Figure 1, and detailed direct effects are reported in Table 3.

**Table 3:** Standardized direct and indirect effects in the structural equation model.

Model paths	β (SE)	p	95% CI lower	95% CI upper
Direct effects				
ConPCR → ST	0.208(0.058)	<0.001	0.094	0.322
CPCR → ST	0.006 (0.048)	> 0.05	−0.088	0.100
ST → IBP	0.148(0.070)	<0.05	0.011	0.285
ST → EBP	0.149(0.068)	<0.05	0.016	0.282
ConPCR → IBP	0.476 (0.089)	<0.001	0.302	0.650
ConPCR → EBP	0.273 (0.079)	<0.05	0.118	0.428
CPCR → IBP	−0.157(0.070)	<0.001	−0.294	−0.020
CPCR → EBP	−0.213 (0.068)	<0.001	−0.346	−0.080
Gender → IBP	0.043(0.039)	> 0.05	−0.033	0.119
Gender → EBP	0.004(0.031)	> 0.05	−0.057	0.065
SES → IBP	−0.076(0.039)	> 0.05	−0.152	0.002
SES → EBP	−0.038(0.031)	> 0.05	−0.099	0.023
Indirect effects (via ST)				
CPCR → ST → IBP	−0.001 (0.008)	> 0.05	−0.017	0.015
CPCR → ST → EBP	−0.001 (0.008)	> 0.05	−0.015	0.018
ConPCR → ST → IBP	0.031 (0.029)	<0.05	−0.026	0.088
ConPCR → ST → EBP	0.031 (0.027)	<0.05	−0.022	0.084

Conflictual parent–child relationship was significantly and positively associated with both internalizing (β = 0.476, *p* < 0.001) and externalizing behavioral problems (β = 0.273, *p* < 0.001), with each standard deviation increase in conflict predicting notable increases in behavioral difficulties. In contrast, close parent-child relationship was negatively associated with internalizing (β= −0.157, *p* < 0.001) and externalizing behavioral problems (β = −0.213, *p* < 0.001). These results underscore the protective role of close parent–child relationships and the risk posed by conflictual interactions in the early development of behavioral problems.

### 4.5 Mediated effect test

Mediation analysis revealed that screen time partially mediated the association between conflictual parent-child relationships and both internalizing and externalizing behavioral problems. Specifically, conflict parent-child relationships predicted higher screen time (β = 0.208, *p* < 0.001), which in turn was associated with increased internalizing (β = 0.148, *p* < 0.001) and externalizing behavioral problems (β = 0.149, p <0.001). Conflict parent–child relationships also had direct effects on internalizing (β = 0.476, *p* < 0.001) and externalizing behavioral problems (β = 0.273, *p* < 0.001). Bootstrapped indirect effects further supported this mediation model: screen time significantly transmitted the effects of conflict on both internalizing (β = 0.031, *p* < 0.05) and externalizing behavioral problems (β = 0.031, *p* < 0.05), confirming a partial mediation pathway. However, no mediation effect was found for the closeness dimension, as its link with screen time was not significant (β = 0.001, *p* > 0.05). This suggests that while parent–child conflict contributes to behavioral difficulties in part through increased screen exposure, the protective effects of relational closeness may operate via alternative mechanisms beyond media use (see Figure 1 and Table 3).

## 5 Discussion

Based on a three-wave longitudinal design, the present study systematically examined the longitudinal associations among parent-child relationships, children's screen time, and behavioral problems in children aged 3 to 6. The findings indicated a temporally ordered pattern of associations: conflictual parent-child relationships at Time 1 were significantly associated with externalizing and internalizing behavioral problems at Time 3, with screen time at Time 2 serving as a partial mediator. In contrast, close parent-child relationships at Time 1 did not significantly predict screen time at Time 2, but were directly and negatively associated with externalizing and internalizing behavioral problems at Time 3. By modeling the distinct pathways of close and conflictual parent-child relationships, the study identified two differentiated patterns of mediation.

### 5.1 Screen time as a partial mediator between conflictual parent–child relationships and behavioral problems

Conflictual parent–child relationships were significantly associated with increased screen time, and screen time partially mediated their association with children's behavioral problems. This pattern is consistent with prior evidence suggesting that high-conflict family environments, particularly those involving criticism, negative affectivity, and communication deficits, may lead children to adopt screen media use as an avoidant coping strategy. In such contexts, screen use may function as an emotion regulation strategy, helping children distance themselves from interpersonal stress and compensate for relational detachment (Konok and Szoke, 2022; Deci and Ryan, 2000). From a family interaction perspective, conflictual parent–child relationships are associated with fewer positive shared activities, including reading, play, and outdoor engagement. Consequently, children may be more likely to engage in screen media for solitary entertainment or time-filling purposes (Poulain et al., 2019; Zhao et al., 2018). Moreover, parent-child conflict may undermine parental regulatory authority over children's media use, potentially increasing screen exposure (Al-Shoaibi et al., 2024).

### 5.2 Close parent-child relationships show no significant effect on children's screen time

The present study found no significant association between close parent–child relationships and children's screen time, which contrasts with prior research suggesting a protective role of relational closeness. One possible explanation is that close relationships are characterized by emotional warmth and acceptance, which may lead parents to adopt more autonomy-supportive practices and fewer restrictive rules regarding media use (Hiniker et al., 2016). Given the inherently engaging nature of screen media and the limited self-regulatory capacity of preschool children (Cliff et al., 2018), children may naturally gravitate toward screen-based activities. Therefore, in the absence of explicit media regulation strategies, relational closeness alone may not be sufficient to reduce screen exposure, nor is it necessarily associated with behavioral adjustment outcomes (Yu et al., 2025; Hu et al., 2018). In some cases, close parent-child relationships embedded in permissive home environments may even coincide with higher screen exposure (Denisenkova and Taruntaev, 2023). This suggests that children's screen time may be more strongly shaped by parental mediation practices than by relational quality alone (Sanders et al., 2018). Even in close relationships, parents may permit limited screen use for educational or recreational purposes due to time constraints, a pattern often described as the “electronic babysitter” phenomenon in Chinese families (Gao et al., 2022). Such practices may weaken the association between relational closeness and screen exposure.

### 5.3 Close parent–child relationship negatively predicts children's behavioral problems

Although close parent–child relationship was not indirectly associated with children's behavioral problems via screen time, it demonstrated a significant direct negative association with both internalizing and externalizing behaviors. This finding aligns with prior research suggesting that close, warm, and supportive relationships enhance children's emotional self-regulation, foster psychological security, and thereby promote the development of prosocial behavior (Chen et al., 2024). Although close parent-child relationships may not directly influence children's screen time, they may support healthier patterns of media use through constructive parental mediation strategies, such as co-viewing and guided interaction (Tu et al., 2024). This may help account for the absence of a significant mediating role of screen time in this pathway.

## 6 Implications and limitations

Amid accelerating digitalization, family caregiving environments and parent–child interaction patterns are undergoing significant transformations. To counteract the developmental risks posed by the increasing reliance on screen media as an “electronic babysitter”, parents are advised to actively engage with their children through meaningful interaction. Such engagement may take the form of shared reading, interactive play, or outdoor physical activities, all of which enrich children's everyday experiences and reduce screen use driven by boredom or inactivity. Moreover, fostering democratic and respectful communication styles helps cultivate emotional closeness and psychological security while minimizing factors like harsh discipline, neglect, emotional distance, and parent-child conflict, which may otherwise strengthen children's emotional reliance on screen media (Domoff et al., 2019). Furthermore, families are encouraged to implement developmentally informed and proactive media intervention strategies, rather than resorting to overly restrictive or permissive approaches. Such strategies should emphasize the prioritization of educational content, the regulation of screen time, and the promotion of active and positive parent-child interactions during media use (Wang et al., 2024). At the same time, parents should also reduce their own screen-focused behaviors and serve as positive role models for media use.

It should be noted that the association between parent–child relationships and children's behavioral outcomes does not necessarily operate directly through screen time. Instead, its underlying mechanisms are likely to be more complex, potentially involving indirect pathways through other mediating or moderating processes (Jusiene et al., 2025). Prior research has suggested that, compared with total screen time, parent–child relationships may exert a stronger influence on the nature and context of children's media use, such as co-viewing vs. solitary use (Rai et al., 2023). Consistent with this perspective, the present findings indicate that although screen time significantly predicts children's behavioral problems, its effect size is relatively modest. Therefore, future research should move beyond a sole focus on screen time duration and further incorporate the content and contextual characteristics of media use. For example, exposure to aggressive or violent content has been associated with an increased risk of externalizing behaviors and socio-emotional difficulties, whereas educational content tends to be associated with comparatively lower developmental risks (Holmgren et al., 2023; Xu et al., 2025). Accordingly, the heterogeneity of media content should be fully considered when interpreting the relationship between screen time and children's behavioral outcomes.

In addition, the present study did not include baseline levels of key variables, which may have resulted in insufficient control for stable between-person differences at initial assessment. As a consequence, some of the observed indirect effects may partially reflect pre-existing levels of the constructs rather than purely developmental processes. If data permit, future research should incorporate baseline measures of both the outcome and mediator variables as autoregressive controls in order to conduct robustness checks. This would allow for a more rigorous identification of dynamic relationships among variables and strengthen the validity of causal inferences. Therefore, caution is warranted when interpreting the causal implications of the observed mediating effects.

Regarding measurement, screen time was assessed using the average duration on weekdays and weekends. Although this approach provides a basic estimate of exposure, it does not capture important contextual information that may be critical for understanding its role in child development. This limitation may have attenuated or obscured the true association between close parent–child interactions and children's behavioral problems (Luo et al., 2024; Bal et al., 2024). Accordingly, future studies should adopt more comprehensive measurement approaches that include not only duration but also content type and usage context.

Regarding the sampling procedure, although participants were recruited through kindergartens and classes, the present study did not explicitly model the nested data structure (e.g., children within classes or schools). Thus, potential clustering effects cannot be entirely ruled out. However, as the analyses focused on individual-level associations, the impact is likely to be limited. Future research may consider accounting for clustering using multilevel approaches or cluster-robust standard errors.

Furthermore, all core variables in the present study were reported by a single informant. Although tests of common method bias indicated that method variance was unlikely to pose a serious threat to the findings, reliance on a single source of information remains a methodological limitation. Incorporating multiple informants would provide a more robust, multi-perspective assessment and further enhance the reliability and validity of the findings. Future research is therefore encouraged to integrate multi-informant data to address this limitation.

Finally, this study employed a short-term longitudinal design. Although this design allows for the examination of temporal ordering among variables, it captures only a limited developmental time window. The relatively short measurement interval may not adequately reflect the dynamic interplay among screen time, parent-child relationships, and behavioral problems over time. Accordingly, the generalizability of the findings across longer developmental periods should be interpreted with caution. Future research should adopt longer-term longitudinal designs with multiple waves of data collection to better capture stability and change in these constructs and to more rigorously test the proposed mediational mechanisms.

## 7 Conclusions

This study sheds light on the dynamic interplay among parent-child relationships, children's screen time, and behavioral problems. The effects of close and conflictual parent-child relationships on screen time diverged, revealing two distinct mediational mechanisms. Conflictual parent-child relationships were a significant predictor of subsequent internalizing and externalizing problems, partially mediated by children's screen time. In contrast, relational closeness was not associated with screen time but exhibited a direct negative predictive effect on externalizing behaviors.

## Data Availability

The original contributions presented in the study are included in the article/supplementary material, further inquiries can be directed to the corresponding author.
